# Augmentation of Polymer-FeCO_3_ Microlayers on Carbon Steel for Enhanced Corrosion Protection in Hydrodynamic CO_2_ Corrosion Environments

**DOI:** 10.1021/acsomega.4c02616

**Published:** 2024-07-09

**Authors:** Dilshad Shaikhah, Wassim Taleb, Maalek Mohamed-Said, Bruce Cowe, Richard Barker

**Affiliations:** † Institute of Functional Surfaces, School of Mechanical Engineering, University of Leeds, Leeds LS2 9JT, United Kingdom; ‡ TotalEnergies, OneTech, CSTJF Avenue Larribau, Pau F-64018, France; § Scientific Research Center, Soran University, Soran, Kurdistan Region 44008, Iraq

## Abstract

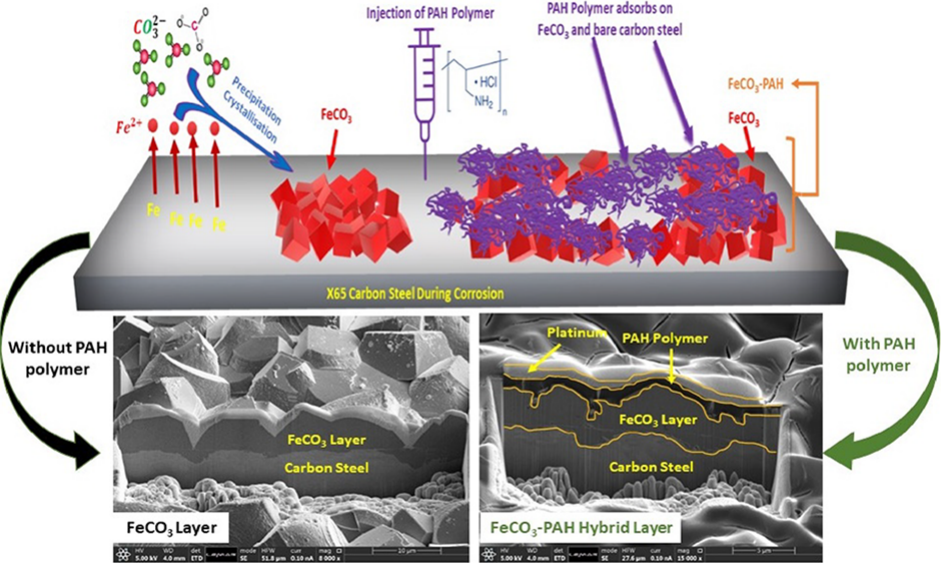

Carbon dioxide (CO_2_) internal corrosion of carbon steel pipelines is a significant challenge and is typically managed by adding corrosion inhibitors. In certain operational conditions, a natural protective layer of iron carbonate (FeCO_3_) can form on the internal walls of the pipeline, offering inhibition efficiency comparable to that of standard surfactant inhibitors. However, incomplete coverage of the FeCO_3_ layer on carbon steel can sometimes trigger localized corrosion. Our previous research demonstrated that poly(allylamine hydrochloride) (PAH) can work synergistically with FeCO_3_ when the corrosion product partially covers X65 carbon steel surfaces in an aqueous CO_2_ corrosion environment. In this study, we utilize rotating cylinder electrode (RCE) tests along with electrochemical measurements to investigate the FeCO_3_–PAH hybrid structure in a dynamic environment. We characterize the general and localized corrosion behavior as well as the surface properties of both naturally formed FeCO_3_ and FeCO_3_–PAH hybrid layers using interferometry and focused ion beam scanning electron microscopy.

## Introduction

Despite the rise of alternative energy sources like renewables and nuclear, fossil fuels continue to be the primary source for meeting global energy needs.^1^ Carbon steel, due to its low cost and wide availability, is the most frequently used material for pipelines in the oil and gas industry, particularly in downhole and upstream operations.^2^ However, carbon steel is prone to internal corrosion, leading to significant economic costs. The expenses related to its degradation and failure account for 10–30% of the maintenance budget in the oil and gas sector.^3^ Therefore, it is crucial to mitigate and control the corrosion of this vulnerable material.

Almost half of the pipeline failures are attributed to either carbon dioxide (CO_2_), so-called sweet corrosion, or hydrogen sulfide (H_2_S), known as sour corrosion.^4^ Controlling corrosion is an expensive protocol based on various strategies proposed by materials and/or corrosion engineers, for instance, modification of materials or solutions,^5,6^ coatings,^7,8^ corrosion inhibitors,^9,10^ and improvements of operations.^11^ Among the aforementioned corrosion control strategies, arguably the most efficient and reliable method to mitigate corrosion is employing corrosion inhibitors owing to the lower relative cost and returns on investments compared to other mitigation techniques.^2^

In CO_2_ and H_2_S corrosion environments, such as those encountered in the geothermal energy, oil and gas, and carbon capture storage industries, various naturally formed corrosion products can play a crucial role in mitigating corrosion. These products act as physical barriers, blocking active corrosion sites and sometimes serving as diffusion barriers to electrochemically active species.^12^ Commonly found layers include magnetite (Fe_3_O_4_),^13^ FeCO_3_ (predominantly forming in CO_2_-containing environments),^14^ and iron sulfides (Fe_*x*_S_*y*_).^15^ These corrosion products can significantly inhibit general corrosion under specific conditions, making it beneficial to maintain them for potential corrosion control. Once these corrosion product layers cover the inner surface of pipelines, they help protect against further corrosion.^14^ However, their protective capability is limited by local heterogeneity or discontinuities in the layer, which can lead to localized corrosion. Additionally, these corrosion products can be removed by mechanical forces (e.g., sand particle impingement) and chemical dissolution.^16−24^

To control the composition, structure, size, and morphology of crystalline minerals in nature, living organisms intercalate organic additives into the inorganic matrix through a process known as biomineralization, resulting in the formation of hybrid materials. These hybrid materials are composed of inorganic and organic constituents on a nano- and microscale, holding distinctive and fascinating physicomechanical properties, which strongly contradict those properties observed by any constituent working independently.^25^ In artificial biomineralization systems, biopolymers have garnered significant attention for the synthesis of mineral hybrids (mainly mineral carbonates) due to their exceptional properties, high compatibility, cost-effectiveness, and adsorption affinity.^26,27^ Among these polymers, ionic polymers stand out as the most promising organic additives because of their solubility in aqueous media, versatile size, and ability to interact with metal carbonates during crystallization.^28^

A representative ionic polymer is poly(allylamine hydrochloride) (PAH), a cationic polymer consisting of amine and ammonium active groups. In our previous studies, this polymer has displayed strong affinity toward carbon steel and the FeCO_3_ corrosion product layer, producing a polymer–corrosion product hybrid layer, which holds distinctive physicomechanical properties, i.e., enhanced adhesion strength, elastic modulus, and resistance to shear stresses, when compared with the natural FeCO_3_ layer.^29,30^

Herein, this study under hydrodynamic CO_2_ corrosion conditions aims to

1. Engineer, *in situ,* a polymer–corrosion product layer consisting of the inorganic FeCO_3_ corrosion product and the organic PAH polymer on X65 carbon steel.
2. Study the crystal structure and morphology of the layers as well as the degree of adsorbed PAH onto FeCO_3_ crystals through the application of various surface analysis techniques.
3. Compare the general corrosion protection of the newly formed hybrid microlayer with pure FeCO_3_ through the application of electrochemical monitoring techniques.
4. Asses the localized corrosion of the surface of the X65 carbon steel specimens in the presence and absence of the PAH polymer.

## Experimental Procedure

### Material Preparation

Given its extensive utilization in the energy industry, carbon steel (API 5L X65) was chosen as the substrate material, serving as the working electrode for electrochemical investigations. Table 1 displays the elemental composition of carbon steel, which exhibits a ferritic–pearlitic microstructure.

**Table 1:** Chemical Composition (wt %) of X65 Carbon Steel^31^

**C**	**Mn**	**Ni**	**Cr**	**Al**	**Mo**	**Si**	**Cu**	**P**	**S**	**Fe**
0.065	1.54	0.04	0.05	0.041	0.007	0.25	0.04	0.013	0.001	balance

**Figure 1:**
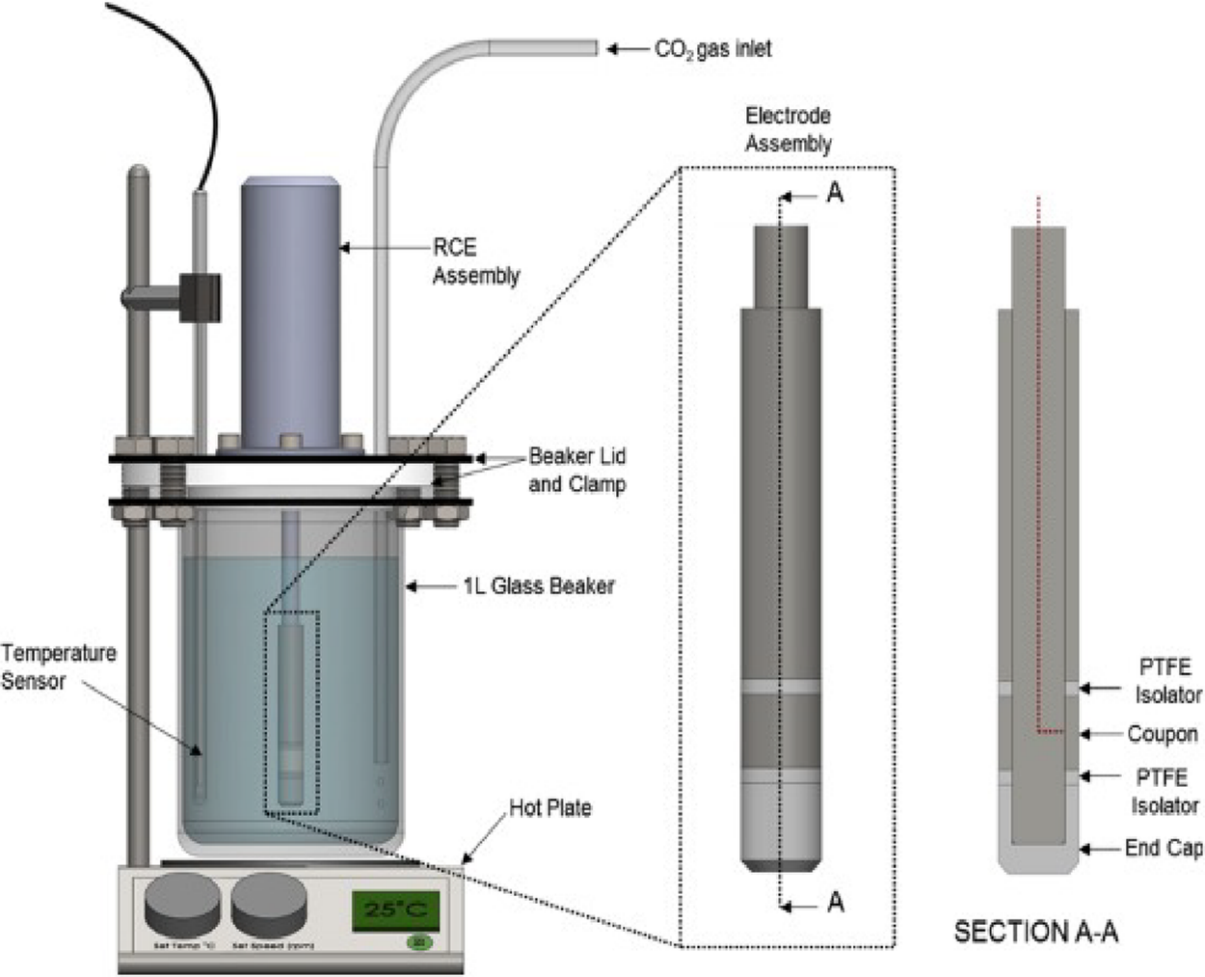
Three-electrode rotating cylinder electrode cell for examining growth of layers in hydrodynamic aqueous CO_2_ environments.^33^

The PAH polymer (average molecular weight: 100,000–150,000) was procured from Alfaeser Scientific and utilized without additional purification. Carbon steel test specimens, serving as working electrodes for electrochemical investigations, were designed into cylindrical tube samples with a surface area of 3.14 cm^2^. As depicted in Figure 1, electrical connections to the working electrodes were established by attaching the cylindrical specimen to the rotator shaft encased with PTFE, exposing a specimen area of 3.14 cm^2^ for corrosion assessments. Subsequently, the exposed surface of each specimen underwent wet grinding up to 600 grit using silicon carbide papers followed by rinsing with deionized water and acetone before drying with nitrogen gas and placement in the aqueous test environment.

### Experimental Methods

Two types of experiments were conducted in this work; first, tests focused on forming a natural FeCO_3_ corrosion layer on carbon steel surfaces, and, second, engineering of a corrosion product–polymer layer was achieved in the presence of a 100 ppm PAH polymer within the aqueous phase. All experiments were conducted using a three-electrode rotating cylinder electrode (RCE) setup, as shown in Figure 1, with some minor differences between each experimental condition, as detailed in the following sections. In all experiments, a 1 L glass beaker was placed on a hot plate to regulate the temperature and was gently stirred using a magnetic stirrer at 500 rpm.

#### FeCO_3_ Layer Growth

The evolution of FeCO_3_ layers has been widely reported on carbon steel surfaces in aqueous CO_2_ environments.^12,32,33^ Conditions in this study were chosen that replicated experiments in which a high surface coverage of FeCO_3_ was achieved on carbon steel at ambient pressure within a 2 day period.^14^ These tests were conducted in hydrodynamic conditions (500 rpm) utilizing an RCE setup integrated with a three-electrode electrochemical cell. A liter of brine solution was prepared by dissolving 35 g of sodium chloride (NaCl) (99% analytical grade, Sigma Aldrich) in 1 L of deionized water for each experiment. The resulting 1 L solution of 3.5 wt % NaCl brine was saturated with CO_2_ overnight by continuous bubbling of CO_2_ gas through the solution. Prior to the commencement of each experiment, the brine solution was heated to 70 °C, and the pH was adjusted to 6.6 by adding sodium bicarbonate (NaHCO_3_) before immersing the prepared carbon steel test specimen into the test solution. The experimental conditions, as outlined in Table 2, were chosen based on previous studies where FeCO_3_formation was observed within a 48 h timeframe.

**Table 2:** Parameters of Hydrodynamic Operations and Polymer Concentrations Employed in the Electrochemical Investigations

parameter	values
rotating rate	500 rpm
temperature	70°C
CO_2_ partial pressure	∼0.07 MPa
substrate material	carbon steel (API 5L X65 steel)
brine medium	3.5 wt % NaCI
pH	6.6 ± 0.20
PAH concentration	100 ppm
time when PAH is first introduced to the medium	2 and 8 h
test duration	48 h

#### FeCO_3_-PAH Hybrid Layer Growth

To investigate the interaction of PAH with the corrosion product on the carbon steel surface as well as the hybrid corrosion protection efficiency, a series of tests focused on in situ engineering of the corrosion product were performed with supporting electrochemical analysis. The PAH polymer itself is cationic and soluble in aqueous solution due to the presence of amine (−NH_2_) and ammonium (−NH_3_^+^) groups in its chemical composition. Initially, the 100 ppm PAH polymer dosage was prepared via dissolving the 100 mg PAH powder into an extracted portion of brine before injecting it back into the solution by a pipette after a fixed precorrosion period to achieve the required dose rate in the 1 L brine solution. This concentration was selected according to our previous studies where the concentration of the PAH polymer was optimized based on static corrosion experiments.^30^

### Electrochemical Approach

LPR tests were carried out to track the corrosion rate over time during the formation of FeCO_3_ and the corrosion polymer–product layer on carbon steel samples. A three-electrode setup was used for these measurements, comprising the carbon steel sample as the working electrode and a combination electrode housing a silver/silver chloride (Ag/AgCl) reference and platinum (Pt) counter electrode, completing the three-electrode configuration. The open circuit potential (OCP) was continuously monitored between LPR measurements, which were conducted every 15 min. During LPR measurements, the carbon steel specimen was subjected to polarization from −15 to +15 mV relative to the OCP, with a scan rate of 0.25 mV/s.

To determine the corrosion rate, the polarization resistance (*R*_p_) in Ω·cm^2^, obtained from the LPR measurements, was used to calculate a corrosion current density (*i*_corr_) in mA/cm^2^:
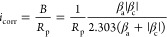
1where *B* is the Stern–Geary coefficient (mV) calculated from β_a_ and β_c_ constants, which are the anodic and cathodic Tafel constants (considered as ±120 mV), respectively. The corrosion rate (*V*_c_) in mm/year is then calculated using Faraday's Law (eq 2):
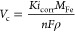
2

Here, *K* represents a conversion factor used to transform the corrosion rate into mm/year (*K* = 3.16 × 105), *M*_Fe_ denotes the molecular mass of iron (55.8 g/mol), *n* stands for the number of electrons liberated in the anodic reaction (*n* = 2), *F* represents the Faraday constant (96,485 C/mol), and ρ indicates the density of the steel (ρ = 7.87 g/cm^3^).

### Surface Analysis

The layers developed on the carbon steel coupons were visualized using Carl Zeiss EVO MA15 scanning electron microscopy (SEM) operating at 20 kV in secondary electron mode. Following corrosion experiments, the coupons underwent carbon coating for SEM examination, capturing top-view images to assess the formed layers.

To investigate the interaction between the PAH polymer and the FeCO_3_ crystal layer, cross sections of carbon steel coupons with FeCO_3_ and FeCO_3_–PAH layers were prepared. These cross sections were processed using an FEI Helios G4 CX DualBeam focused ion beam (FIB)-SEM to examine the steel/layer interface. The specimens were oriented at 52°, carbon-coated, and imaged in secondary electron mode at 5 kV before depositing a platinum layer. Subsequently, a gallium ion beam was employed to mill a 15 μm wide, 10 μm deep cross section at an operating current of 21 nA. A series of cross-sectional cleaning steps ensued at an operating current of 2.5 nA. Post-preparation, gallium beam images of the cross sections were captured at an operating current of 7.7 pA. These FIB analysis images were utilized to measure the thickness of the corrosion and polymer–corrosion layers.

#### Surface Profilometry Analysis and Localized Corrosion Measurement

The assessment of localized corrosion on the X65 carbon steel surface utilized a surface profilometry method, employing the 3D surface profilometer Bruker NPFlexTMiv. Preceding analysis, the specimens post-test underwent preparation by surface cleaning using Clarke’s solution following ASTM Standard G 1-03.^34^ For each experimental condition, seven regions were scanned, covering a total area of 49 mm^2^ (7 × 7 mm^2^) per specimen. Subsequent analysis of the raw data was conducted using the Vision64 software package, employing specific thresholds (5 μm pit depth) to meticulously quantify pit depths, diameters, and areas, consistent with prior research.^35^

## Results and Discussion

### Augmentation and Corrosion Behavior of the FeCO_3_ Layer and FeCO_3_–PAH Hybrid Layer

**Figure 2:**
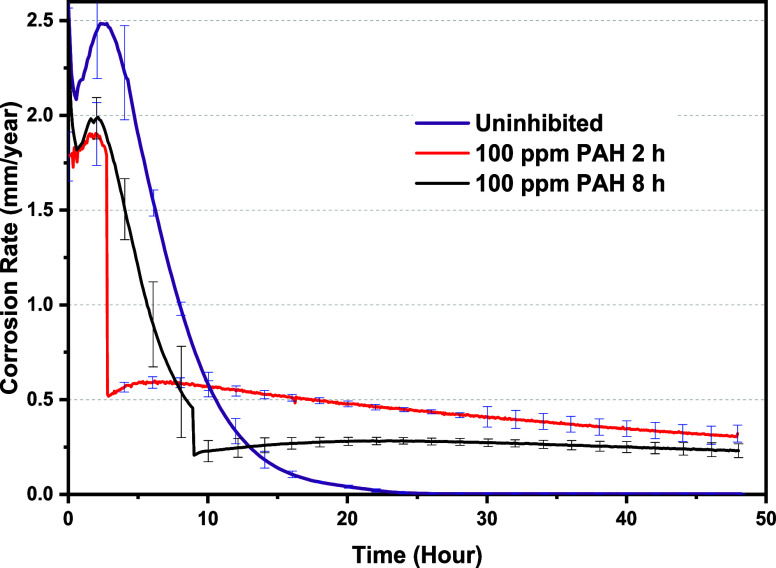
Corrosion rates over time for X65 carbon steel in hydrodynamic conditions (500 rpm, pH 6.6, 70 °C, 3.5% NaCl) for 2 days. Includes natural FeCO_3_ growth and response to 100 ppm of PAH added at 2 and 8 h.

The corrosion rates observed in the hydrodynamic experiments, where either an FeCO_3_ layer or FeCO_3_-PAH hybrid layer formed on the carbon steel surface, are depicted in Figure 2, plotted against time. The average corrosion rates reported are based on a minimum of three repeated experiments.

To evaluate the effect of the PAH polymer on corrosion behavior and FeCO_3_ growth under hydrodynamic conditions, a 100 ppm dosage was introduced to separate cells after 2 and 8 h of precorrosion for 2 days. At rotation rates of 500 rpm (as shown in Figure 2), in the absence of the PAH polymer (labeled as uninhibited), the corrosion rate initially measures 2.5 mm/year, progressively decreasing to less than 0.1 mm/year after 20 h of corrosion and further dropping to 0.01 mm/year by the end of the experiment.

Upon injecting 100 ppm of the PAH polymer into the solution at 2 h of precorrosion, the corrosion rate decreases to 0.5 mm/year in less than 5 min. This suggests an immediate interaction of the PAH polymer with the bare carbon steel surface, where no corrosion product layer is formed. The corrosion rate then gradually reduces to below 0.3 mm/year by the end of the 2 day period. Additionally, injecting PAH polymer at an 8 h precorrosion reduces the corrosion rate from 0.5 to 0.2 mm/year, indicating an impact on corrosion kinetics, indirectly suggesting an interaction between the polymer and both the steel surface and corrosion product layer. This aligns with the PAH corrosion efficiency observed in static conditions when the precorrosion time was extended to 24, 34, and 44 h.^29,30^

Generally, the corrosion measurements in Figure 2 suggest that the PAH interferes with the growth of the FeCO_3_, and this could be through reducing Fe^2+^ flux into the solution, not necessarily through direct interaction of the crystals with the PAH. However, the fact that the CR drops more when FeCO_3_ exists on the surface suggests that there may be some interaction between PAH and preformed FeCO_3_ crystals. Furthermore, the corrosion rate response implies that the PAH polymer added at 2 and 8 h of precorrosion affects the crystal growth of FeCO_3_ on the surface of carbon steel as the evolution of the saturation ratio is now shifted due to the PAH corrosion inhibition decreasing the flux of Fe^2+^ into the solution. This is discussed further in the Surface Morphology and Analysis section. The general residual corrosion rate for polymer added at 2 and 8 h of precorrosion drops gradually to 0.25 mm/year, which is higher than the corrosion rate residual in the absence of the polymer, which is 0.01 mm/year. However, the localized corrosion is reduced severely when the PAH polymer is introduced into the corrosion environment; this is discussed in detail in the Localized Corrosion Mapping of the Pure FeCO_3_ and Hybrid FeCO_3_ Systems section. As the corrosion rate is still above 0.1 mm/year, there is the potential for FeCO_3_ formation; however, this is much slower compared with the uninhibited conditions.

### Surface Morphology and Analysis

**Figure 3:**
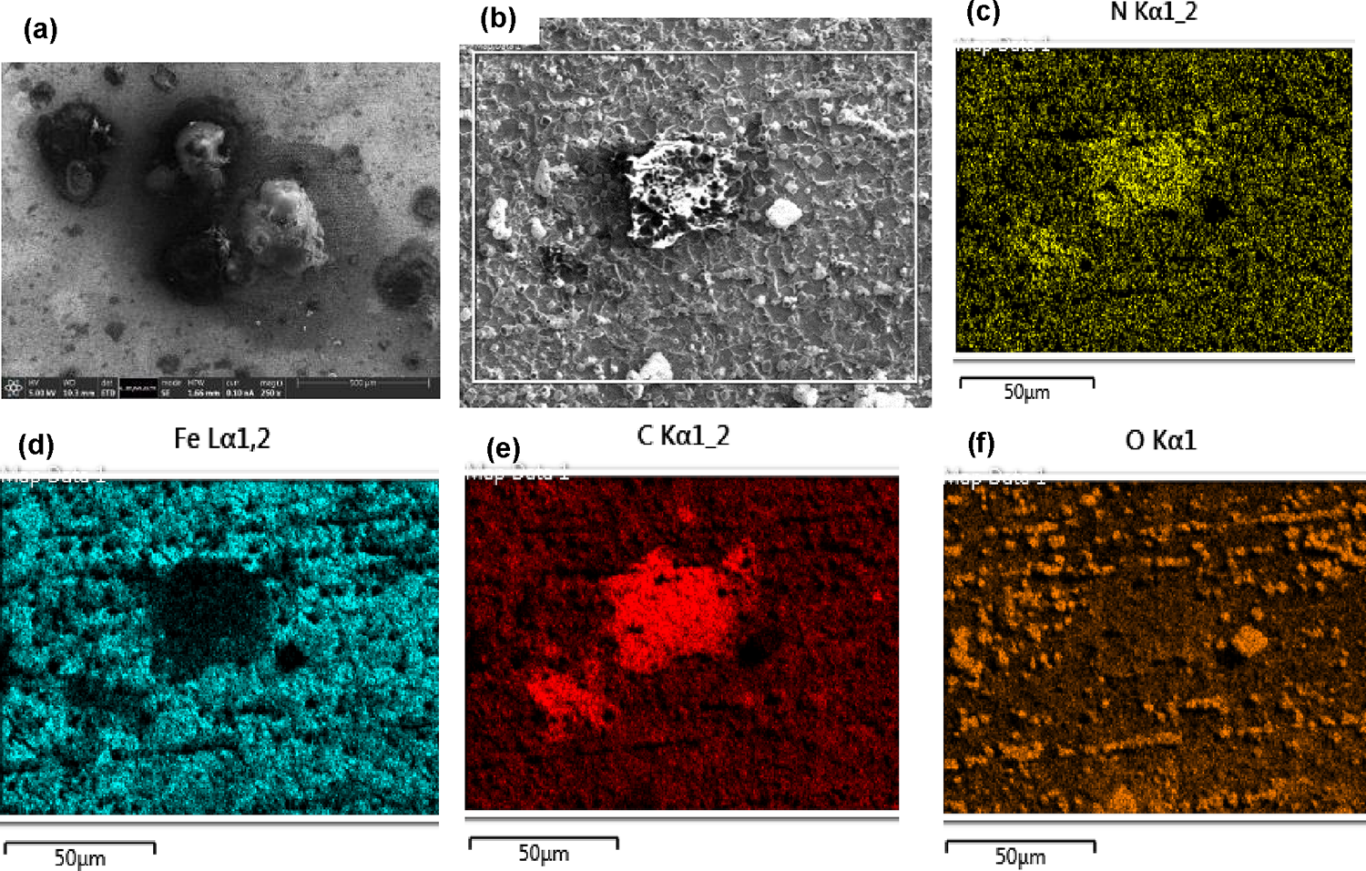
SEM-EDX mapping of the FeCO_3_–PAH hybrid layer on X65 steel after 100 ppm PAH injection at a 2 h precorrosion in a CO_2_ saturated environment for 48 h. (a, b) Surface, (c) nitrogen map, (d) Fe map, (e) carbon map, and (f) oxygen map (conditions: 500 rpm, pH 6.6, 70 °C, 3.5 wt % NaCl).

Surface characterization shows that the morphology and extent of FeCO_3_ crystals in the presence of the PAH polymer are different compared with the crystal structure of FeCO_3_ grown naturally, indicating the impact of polymer molecules on the size and morphology of FeCO_3_ crystals. SEM-EDX analysis was employed to examine the composition and structure of the FeCO_3_–PAH hybrid layer. Figure 3 displays an SEM micrograph of a selected region and its elemental mapping for the specimen exposed to 100 ppm PAH after 48 h with a 2 h precorrosion period. Observations from the carbon mapping micrograph revealed that the PAH polymer coats the carbon steel surface, including FeCO_3_ crystals. Additionally, the presence of FeCO_3_ beneath the PAH polymer is confirmed by the mapping of iron and oxygen, as depicted in Figure 3.

**Figure 4:**
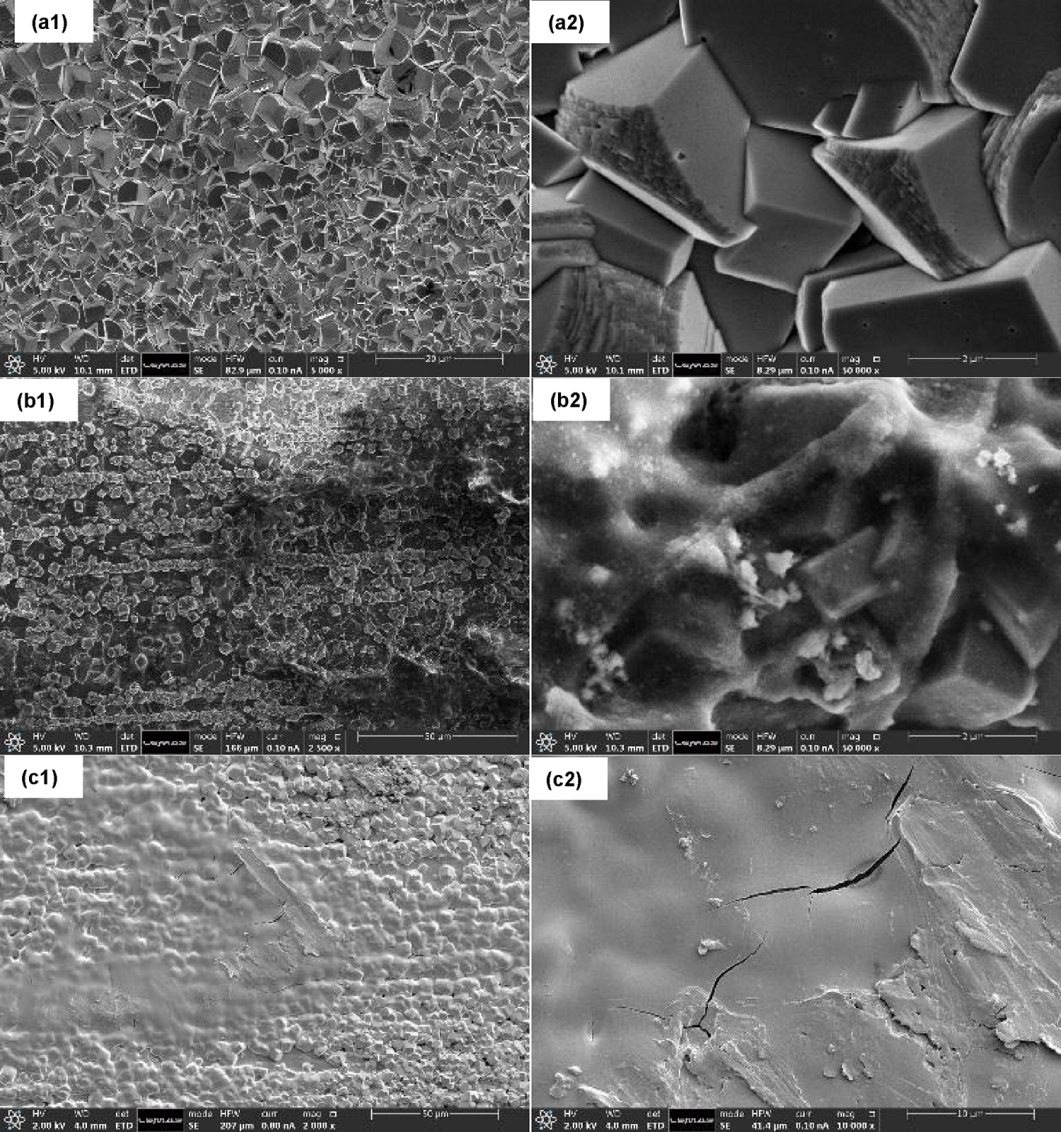
SEM images of steel surfaces after 48 h in (a1, a2) uninhibited and with the PAH polymer added at (b1, b2) 2 h and (c1, c2) 8 h of precorrosion: 100 ppm PAH. Conducted in CO_2_-saturated conditions (500 rpm, pH 6.6, 3.5 wt % NaCl, 70 °C).

Comparison of SEM images in Figure 4 illustrates the difference between carbon steel coupons coated with FeCO_3_ alone and those with FeCO_3_–PAH hybrid layers formed at pH 6.6, with 100 ppm of the PAH polymer introduced after a 2 h precorrosion period. Without the PAH polymer, the carbon steel surface (Figure 4a1,a2) exhibits a complete FeCO_3_ layer. However, with the addition of the PAH polymer, less FeCO_3_ is observed on the surface, along with the adsorbed polymer (Figure 4b1,b2,c1c2). Injection of 100 ppm of the PAH polymer results in a partially covered surface with smaller FeCO_3_ crystals and PAH polymer combination (Figure 4b1,b2,c1,c2), compared to the uninhibited condition.

Previous experiments in a static environment revealed the interaction of the PAH polymer with FeCO_3_ crystals and the bare steel surface due to its active amine and ammonium centers.^29^ Similar to the static environment, the PAH polymer in the hydrodynamic environment is adsorbed not only onto the FeCO_3_ crystals but also on the bare steel surface, filling the gaps between the crystals (Figure 4b2). Various studies have established that the presence of heterogeneity or discontinuity among the corrosion products induces pitting and galvanic corrosion limiting the corrosion product exploitation as a mitigation approach.^16−24^ Nonetheless, images in Figure 4b show the uniform coverage of the PAH polymer on the steel surface. This implies that the PAH reduces the heterogeneity or discontinuity within the FeCO_3_ crystal layer via interconnection of such crystals, producing a hybrid layer, which can more readily be exploited for enhanced corrosion protection.

**Figure 5:**
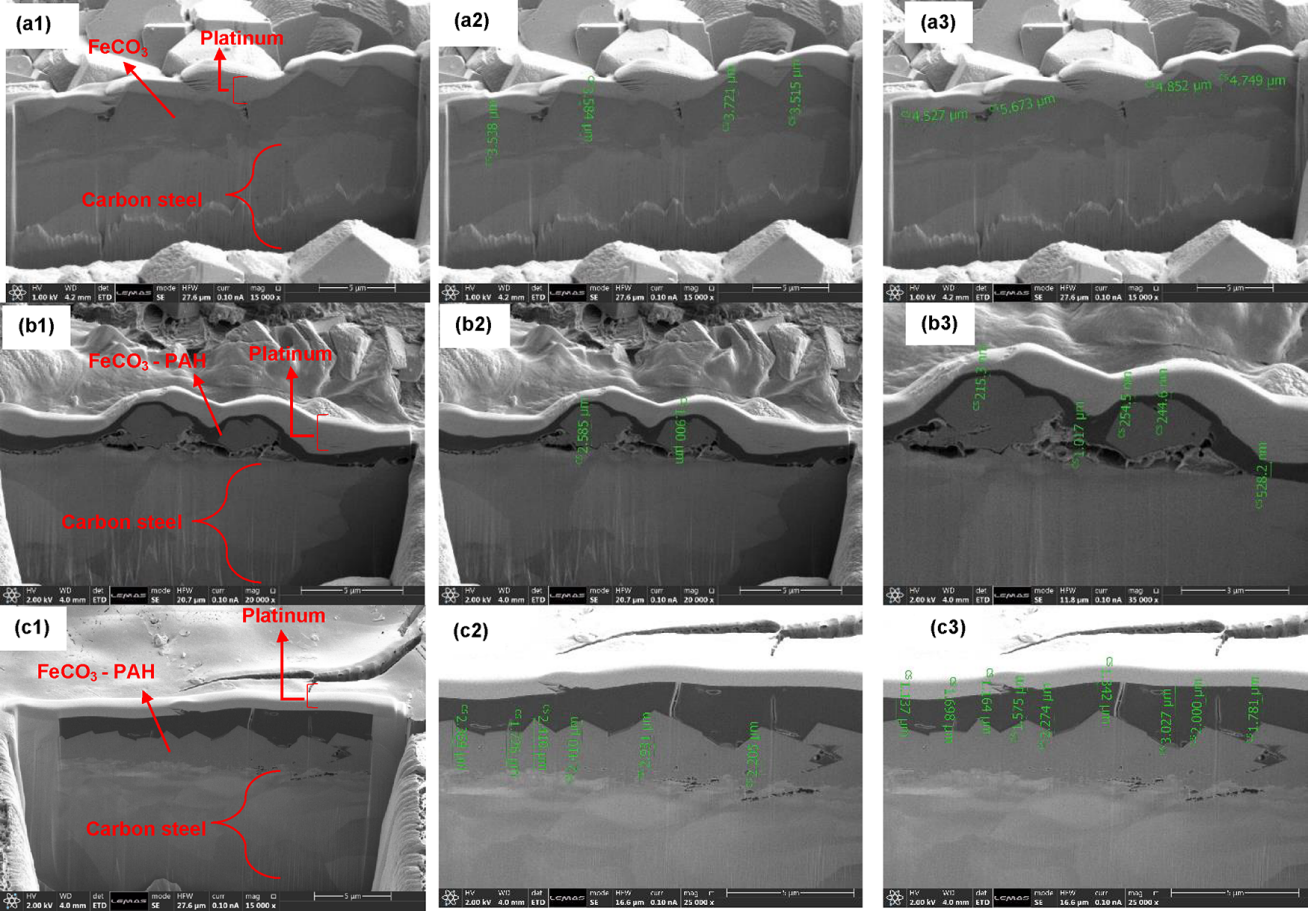
SEM-FIB cross-sectional images of carbon steel after 48 h in the electrolyte (500 rpm, pH 6.6, 70 °C, 3.5 wt % NaCl, pCO_2_). Images (a1–a3) show the absence of PAH, while images (b1–b3, c1–c3) depict 100 ppm PAH at 2 and 8 h of precorrosion, respectively.

The results obtained from the FIB-SEM analysis provided valuable insights into the thickness of the deposited layers and the growth process of the hybrid film. The SEM images presented in Figure 5 offer a comparative view between the uninhibited system and the systems where the PAH polymer was introduced after different precorrosion times.

In Figure 5a, representing the uninhibited system, we observe substantial coverage of the FeCO_3_ crystalline layer on the carbon steel surface. However, Figure 5b and Figure 5c, which depict the systems with the PAH polymer introduced after 2 and 8 h of precorrosion, respectively, reveal a different scenario. Here, we notice a distinct PAH polymer layer covering individual crystals, indicating an interaction between the polymer and both the carbon steel surface and FeCO_3_ crystals. This interaction leads to the formation of an FeCO_3_–PAH hybrid layer, as evidenced by the SEM images.

Further analysis of the layer thickness reveals significant differences between the systems with and without the PAH polymer. Without the polymer, the FeCO_3_ crystal layer thickness ranges from 3 to 6 μm. However, with the introduction of the PAH polymer after 2 h of precorrosion, this thickness reduces to around 1 μm, and it further decreases to 2.5 μm when the polymer is added after 8 h of precorrosion. Additionally, the FeCO_3_–PAH hybrid layers exhibit crystal thicknesses between 1 and 1.5 μm and top adsorbed PAH polymer thicknesses ranging from 250 to 1000 nm. Notably, a higher thickness of the PAH polymer (2 μm) is observed when added at 8 h of precorrosion.

The presence of FeCO_3_ fine crystals beneath the PAH polymer layer suggests an interesting dynamic in the nucleation and growth processes, akin to observations made during calcite nucleation and crystal growth. This finding underscores the complex interplay between the PAH polymer and FeCO_3_ crystals, influencing the overall corrosion process.

Moreover, the adsorption of the PAH polymer on both the carbon steel surface and the FeCO_3_ crystal layer serves as a physical hydrophobic barrier. This barrier effectively segregates the steel surface from the corrosive media, disrupting the transfer of ferrous ions (Fe^2+^) and slowing the nucleation and growth of FeCO_3_. This mechanism highlights the potential of the PAH polymer in mitigating corrosion by altering the corrosion kinetics and forming a protective barrier against further corrosion processes.

### Localized Corrosion Mapping of the Pure FeCO_3_ and Hybrid FeCO_3_ Systems

**Figure 6:**
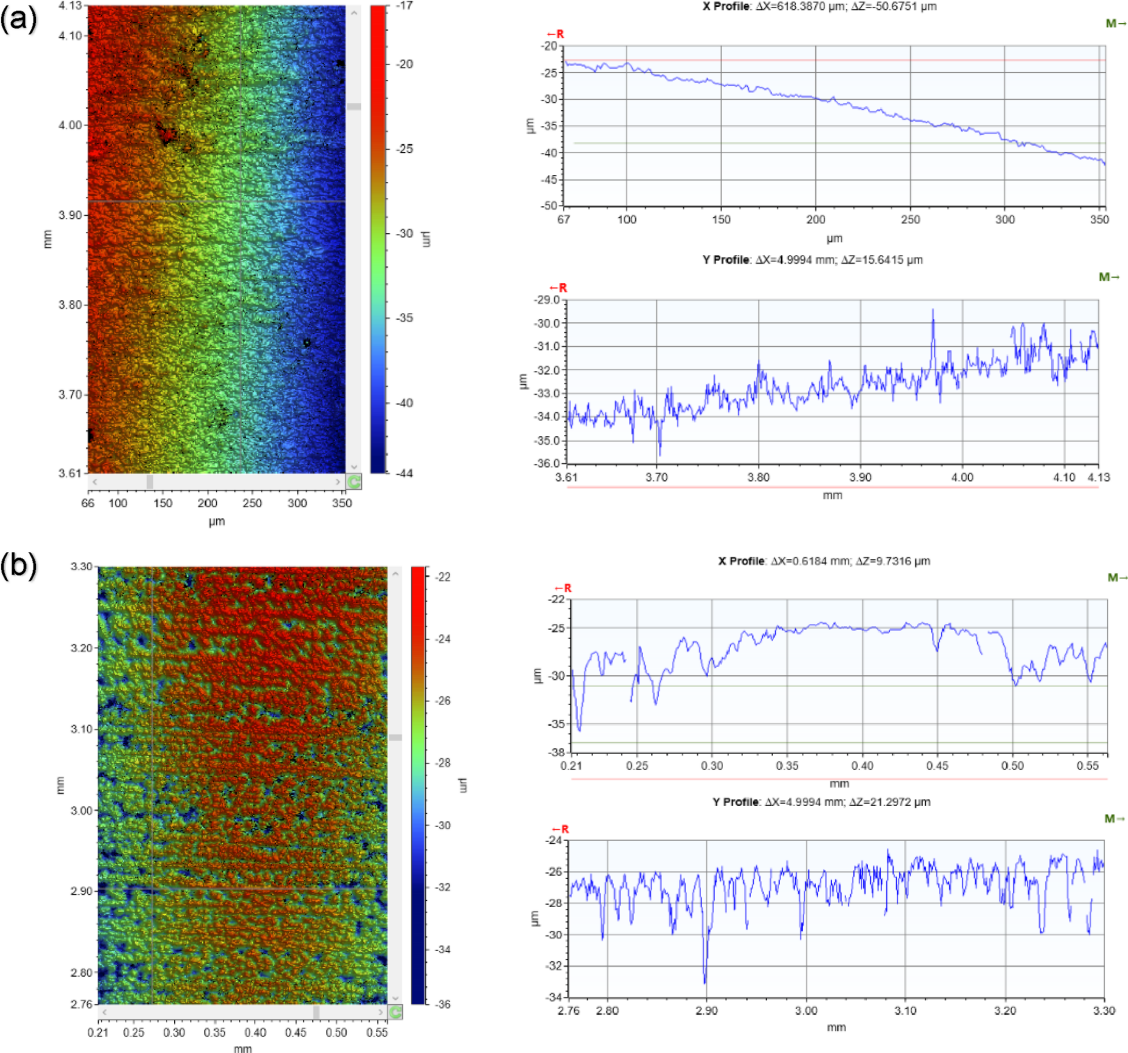
Vertical light interferometry 2D images of the deepest pits for (a) FeCO_3_ and (b) FeCO_3_–PAH hybrid layer (500 rpm, pH 6.6, 70 °C, 3.5 wt % NaCl, pCO_2_, 0.07 MPa).

The adsorption behavior of PAH suggests that the incorporation of the polymer could alter the morphology and thickness of the corrosion layer, potentially enhancing its protective properties against both general and localized corrosion. Figure 6 illustrates the impact of the PAH polymer on localized corrosion rates after 48 h, following injection at a 2 h precorrosion stage.^35−37^ Alongside mitigating uniform corrosion of the steel surface, the PAH polymer significantly reduces the rate of localized corrosion and the formation of surface pits. Table 3 presents localized corrosion measurements (10 deepest pits) for both uninhibited and PAH polymer corrosion environments. Results indicate that in the uninhibited system, pit depth and diameter range from 108 to 80 μm. Conversely, the addition of the PAH polymer results in a substantial decrease in pit depth by a factor of 100 as well as a reduction in the average diameter of pits. Furthermore, steel surface roughness decreases from 5.65 to 1.24 μm after 48 h when the PAH polymer is injected 2 h prior to corrosion. This reduction in roughness can be attributed to the synergistic inhibition effect of the PAH polymer, which adsorbs onto the metallic surface and interacts with corrosion products present. Additionally, a recent study investigated the impact of imidazoline-type inhibitors on localized corrosion after a 2 h precorrosion period for carbon steel in a CO_2_-saturated environment.^38^

**Table 3:** Ten Deepest Pits and Their Diameters Measured by Light Interferometry for Experiments with and without 100 ppm PAH Added after 2 h of Exposure^a^

**uninhibited**		**100 ppm PAH at 2 h**	
pit depth (μm)	pit diameter (μm)	pit depth (μm)	pit diameter (μm)
107.9	49.6	12.0	9.7
107.5	6.2	10.6	33.0
103.6	29.4	10.6	34.9
94.0	23.9	10.0	33.6
92.5	3.5	9.3	11.3
92.1	26.3	9.0	10.8
89.5	16.6	8.8	34.6
88.6	47.8	8.4	28.7
88.1	11.8	8.4	36.1
79.9	13.9	8.3	28.9

a 500 rpm, pH 6.6, 70°C, 3.5 wt % NaCl, pCO_2_ 0.07 MPa, 48 h.

When corrosion products coat the steel surface, traditional imidazoline surfactants used for CO_2_ corrosion inhibition often exhibit inadequate protection against general corrosion and may even exacerbate localized corrosion. The findings indicate that localized corrosion tends to increase with the addition of imidazoline inhibitors at the 2 h immersion mark, whereas the introduction of the PAH polymer reduces localized corrosion.

Notably, introducing the PAH polymer at the 2 h exposure stage, when fewer FeCO_3_ crystals are present on the metal surface, leads to shallower pits, as depicted in Figure 6, resulting in a less aggressive localized attack. These observations regarding the PAH polymer are intriguing, considering that many studies on more conventional CO_2_ corrosion inhibitors suggest that precorrosion and the formation of corrosion products have an adverse effect on localized corrosion.^38−40^

For instance, a recent study by Shamsa et al. examined the impact of imidazoline-type inhibitors on localized corrosion after a 2 h precorrosion period for carbon steel in a CO_2_-saturated environment.^38^ The findings indicated an increase in localized corrosion when the imidazoline inhibitor was added at the 2 h mark, whereas the PAH polymer reduced localized corrosion.

To optimize the dosage and precorrosion timing of the PAH polymer concerning localized corrosion and galvanic interactions, further investigation into the mechanisms of localized corrosion retardation and galvanic corrosion is warranted. Ultimately, experiments conducted at the 2 h mark and over longer durations are necessary to assess the influence of PAH on pit growth and retardation, determining the optimal timing for injecting PAH polymer to mitigate localized corrosion.

## Conclusions

Inspired by biomineralization processes, we have demonstrated the potential for specifically selected chemicals to interact/adsorb favorably with FeCO_3_ corrosion products on steel surfaces as well as the steel substrate itself in CO_2_-containing corrosive environments. This was achieved using PAH, a cationic polymer, which produces a hybrid layer providing enhanced general and localized corrosion protection in contrast to other chemistries that fail to work effectively in the presence of corrosion products. From this study, it is possible to conclude that

- The CO_2_ general and localized corrosion kinetics of carbon steel were dramatically reduced by the PAH polymer when 100 ppm was administered to the corrosion environment.
- This was attributed to the PAH polymer exhibiting a synergistic impact, adsorbing onto the bare steel surface substrate as well as interacting favorably with FeCO_3_ crystals to produce a more effective inhibition layer.
- Even though the remaining general corrosion rate was higher when the PAH polymer was present, compared to a completely formed “pure” FeCO_3_ layer, the addition of the PAH polymer was significantly more effective in reducing localized corrosion.
- Besides the reduction in the general corrosion rate by the newly developed FeCO_3_–PAH hybrid layer compared to a pure FeCO_3_ layer, the hybrid layer offers superior localized corrosion defense in comparison to other chemical compositions in similar CO_2_-rich conditions. More comprehensive testing is necessary to accurately identify and measure these advantages.
